# Hepatitis E Virus Infection, Papua New Guinea, Fiji, and Kiribati, 2003–2005

**DOI:** 10.3201/eid2006.130562

**Published:** 2014-06

**Authors:** John S. Halliday, G.L. Abby Harrison, Anthony Brown, Jeremy G. Hunter, Richard Bendall, David Penny, Tebuka Toatu, Mohammad Y. Abdad, Paul Klenerman, Eleanor Barnes, Harry R. Dalton

**Affiliations:** University of Oxford, Oxford, UK (J.S. Halliday, A. Brown, P. Klenerman, E. Barnes);; Royal Melbourne Hospital, Melbourne, Victoria, Australia (J.S. Halliday);; Walter and Eliza Hall Institute of Medical Research, Melbourne (G.L.A. Harrison);; University of Exeter, Truro, UK (J.G. Hunter, R. Bendall, H.R. Dalton);; Massey University, Palmerston North, New Zealand (D. Penny);; Disease Surveillance, Control and Research Unit, Secretariat of the Pacific, Suva, Republic of the Fiji (T. Toatu);; Papua New Guinea Institute of Medical Research, Goroka, Papua New Guinea (M.Y. Abdad)

**Keywords:** Hepatitis E, seroprevalence, South Pacific, infection, viruses, Papua New Guinea, Fiji, Kiribati, zoonoses

**To the Editor:** We report hepatitis E virus (HEV) infection rates in 3 South Pacific island countries—Papua New Guinea (PNG), Fiji, and Kiribati—determined from results of HEV IgG testing. During 2003–2005, specimens were collected from volunteers as part of a study of the epidemiology of viral hepatitis (*1*,*2*). Participants recruited were apparently healthy adults in the community and mother–infant pairs (specifically infants who were receiving, or had recently completed, their vaccinations). No specific inclusion/exclusion criteria were applied. Samples were collected from outpatient clinics and hospitals in PNG from Port Moresby, Goroka, Mt Hagen, Madang, and Daru. Samples from Fiji were collected in Suva from outpatient clinics and hospital wards. A proportion of samples from children were taken from nonjaundiced inpatients in PNG and Fiji. In Kiribati, samples were collected from participants at village preschools, vaccination clinics, and outpatient clinics on North Tarawa and North Tabiteuea (*2*). These were convenience samples and therefore might not be nationally representative cohorts. We obtained ethics permission for the study from appropriate national agencies. Signed informed consent was obtained from each participant or, for children, from a parent or guardian.

From this sample pool, in a time sequential manner, the first serum samples were assayed: 545 from PNG (48 Goroka, 99 Mt Hagen, 87 Daru, 47 Madang, 156 Port Moresby), 265 from Fiji, and 238 from Kiribati. We evaluated samples using the Wantai (PE2) HEV IgG ELISA kit (Wantai Pharmaceutical Enterprise, Beijing, China), which detects IgG for all 4 known strains of human HEV. The assays were used according to the manufacturer’s instructions, and repeat equivocal results were defined as negative (*3*,*4*). HEV IgG positivity was highest in PNG (15.2%), followed by Kiribati (8.8%) and Fiji (2.2%) (Table). IgG positivity did not differ significantly between adults and children (<16 years of age) (PNG: 16.1% vs. 11.4%, p = 0.23; Kiribati: 6.0% vs. 13.3%, p = 0.06; and Fiji: 1.7% vs. 3.3%, p = 0.42 [Fisher exact test]).

**Table:** Seroprevalence of HEV, Papua New Guinea, Fiji, and Kiribati, 2003–2005*

Age group, y	Papua New Guinea†			Fiji†			Kiribati†		
	All	HEV IgG+, % (95% CI)		All	HEV IgG+, % (95% CI)		All	HEV IgG+, % (95% CI)
Children	12/105‡	11.4 (6.6–18.9)		3/91‡	3.3 (1.1–9.2)		12/90	13.3 (7.8–21.2)
<1	0/21	0		1/23	4.3		1/8	12.5
1–<2	3/26	11.5		1/19	5.3		2/18	11.1
2–<5	6/24	25		1/35‡	2.9		6/48	12.5
5–<10	1/23‡	4.3		0/10‡	0		0/10	0
10–<16	1/6	16.7		0/3‡	0		0/2	0
Unknown	1/5	20		0/1	0		3/4	75
Adults	71/440	16.1 (14.2–21.3)		3/174	1.7 (0.6–4.9)		9/148	6.0 (3.2–11.1)
16–19	7/24	29.2		0/8	0		0/2	0
20–29	19/162	11.7		2/76	2.6		2/61	3.3
30–39	18/143	12.6		0/41	0		3/53	3.7
40–49	13/70	18.6		1/28	3.6		2/19	5.3
50–59	5/22	22.7		0/14	0		2/9	22.2
60–69	3/7	42.9		0/5	0		0/2	0
70–79	0/4	0		0/0	0		0/2	0
Unknown	0/9	0		0/1	0		0/0	0
Total	83/545‡	15.2 (12.5–18.5)		6/265‡	2.3 (1.0–4.9)		21/238	8.8 (5.8–13.1)

*An expanded version of this table that includes sex data is available online (wwwnc.cdc.gov/EID/article/20/7/13-0562-T1.htm). HEV, hepatitis E virus; +, positive. †No. HEV IgG–positive persons/total no. persons in each group. ‡In these groups.

To investigate potential parent–child transmission, we tested mother/child (MC); father/child (FC); and when possible, mother/father/child (MFC) sets. We found no transmission association: In PNG, we tested 88 sets (67 MC, 2 MFC, and 19 FC); in Fiji, 29 sets (20 MC and 9 MFC); and in Kiribati, 65 sets (59 MC and 6 MFC); of the 11 PNG, 1 Fijian, and 8 Kiribati HEV IgG–positive children, none had IgG-positive parents. Because these samples were tested retrospectively, ascertaining the HEV IgG status of other family members was not possible.

The high percentage of HEV-seropositive children <5 years of age in PNG and Kiribati implies active viral circulation in these countries. This is an unusual finding, compared with findings from seroprevalence studies in developing countries where IgG prevalence increases with age (*3*). The reason for this difference remains to be determined. It is unlikely to relate to acute HEV infection in hospitalized children sampled because the Wantai assay measures IgG, not IgM. The finding that these young seropositive children commonly have seronegative parents suggests that parent–child transmission is not the primary mechanism of infection in the population studied, which is in accord with published data (*4*).

To investigate whether HEV seropositivity was higher in certain areas, we partitioned the data into regions defined by participant’s place of birth and tribal ethnicity. In Fiji and Kiribati, no significant region association was detected. However, in PNG, the proportion of HEV antibody–positive specimens was greater among participants from highland communities (altitude >1,500 m) than from lowland communities (20.4% vs. 9.7%, p = 0.01). (Port Moresby has a mixed immigrant population and was excluded from this analysis).

The reason for the higher proportion of HEV IgG–positive specimens among participants in highland than lowland communities is unclear but might be explained by increased zoonotic transmission. In highland regions, pigs are more frequently kept, and the animals are kept closer to home (*5*). HEV genotypes 1 and 2 are hyperendemic to many developing countries and typically cause waterborne outbreaks of acute hepatitis in humans (*6*). Genotypes 3 and 4 are endemic to industrialized countries and are known to be a porcine zoonosis (*7*). Genotype 3 has been found in pigs in New Caledonia (*8*).

In our investigation of 3 developing nations in Oceania, we found that HEV IgG positivity varies substantially between, and within, countries; it is high in PNG (15.2%) and low in Fiji (2.2%). By using the same sensitive, diagnostic assay, the seroprevalence of HEV in blood donors in New Zealand was reportedly 4% (*9*). In New Caledonia, 1.7% of 351 military recruits tested positive (*10*). In our study, the sampling method limits the applicability of the data to the general population. Nevertheless, our findings suggest HEV infection should be considered in cases of unexplained hepatitis.
